# Long noncoding RNA repertoire in chicken liver and adipose tissue

**DOI:** 10.1186/s12711-016-0275-0

**Published:** 2017-01-10

**Authors:** Kévin Muret, Christophe Klopp, Valentin Wucher, Diane Esquerré, Fabrice Legeai, Frédéric Lecerf, Colette Désert, Morgane Boutin, Frédéric Jehl, Hervé Acloque, Elisabetta Giuffra, Sarah Djebali, Sylvain Foissac, Thomas Derrien, Sandrine Lagarrigue

**Affiliations:** 1 UMR PEGASE, INRA, 35042 Rennes, France; 2 UMR PEGASE, AGROCAMPUS OUEST, 35042 Rennes, France; 3 SIGENAE, INRA, 31326 Castanet-Tolosan, France; 4 UMR6290 IGDR, CNRS, Université Rennes 1, 35000 Rennes, France; 5 Plateforme GENOTOUL, INRA, 31326 Castanet-Tolosan, France; 6 GenPhySE, INPT, ENVT, INRA, Université de Toulouse, 31326 Castanet-Tolosan, France; 7 UMR IGEPP, INRA, 35042 Rennes, France; 8 UMR IGEPP, AGROCAMPUS OUEST, 35042 Rennes, France; 9 GABI, AgroParisTech, INRA, Université Paris Saclay, 78350 Jouy-en-Josas, France

## Abstract

**Background:**

Improving functional annotation of the chicken genome is a key challenge in bridging the gap between genotype and phenotype. Among all transcribed regions, long noncoding RNAs (lncRNAs) are a major component of the transcriptome and its regulation, and whole-transcriptome sequencing (RNA-Seq) has greatly improved their identification and characterization. We performed an extensive profiling of the lncRNA transcriptome in the chicken liver and adipose tissue by RNA-Seq. We focused on these two tissues because of their importance in various economical traits for which energy storage and mobilization play key roles and also because of their high cell homogeneity. To predict lncRNAs, we used a recently developed tool called FEELnc, which also classifies them with respect to their distance and strand orientation to the closest protein-coding genes. Moreover, to confidently identify the genes/transcripts expressed in each tissue (a complex task for weakly expressed molecules such as lncRNAs), we probed a particularly large number of biological replicates (16 per tissue) compared to common multi-tissue studies with a larger set of tissues but less sampling.

**Results:**

We predicted 2193 lncRNA genes, among which 1670 were robustly expressed across replicates in the liver and/or adipose tissue and which were classified into 1493 intergenic and 177 intragenic lncRNAs located between and within protein-coding genes, respectively. We observed similar structural features between chickens and mammals, with strong synteny conservation but without sequence conservation. As previously reported, we confirm that lncRNAs have a lower and more tissue-specific expression than mRNAs. Finally, we showed that adjacent lncRNA-mRNA genes in divergent orientation have a higher co-expression level when separated by less than 1 kb compared to more distant divergent pairs. Among these, we highlighted for the first time a novel lncRNA candidate involved in lipid metabolism, lnc_DHCR24, which is highly correlated with the *DHCR24* gene that encodes a key enzyme of cholesterol biosynthesis.

**Conclusions:**

We provide a comprehensive lncRNA repertoire in the chicken liver and adipose tissue, which shows interesting patterns of co-expression between mRNAs and lncRNAs. It contributes to improving the structural and functional annotation of the chicken genome and provides a basis for further studies on energy storage and mobilization traits in the chicken.

**Electronic supplementary material:**

The online version of this article (doi:10.1186/s12711-016-0275-0) contains supplementary material, which is available to authorized users.

## Background

Long noncoding RNAs (lncRNAs) are commonly defined as non protein-coding transcripts that are often spliced, capped and polyadenylated but have little or no protein-coding potential. Genome-wide transcriptional studies carried out by ENCODE (Encyclopedia of DNA Elements) and other large international consortia [1] have revealed that more than 60% of mammalian genomes are transcribed and that a large fraction of the transcripts is represented by lncRNAs [1–5]. Among these studies, the GENCODE consortium has collated a comprehensive set of human lncRNAs and analyzed their genomic organization, modifications, cellular locations and tissue expression profiles in different human cell lines [6].

Since 2012, the number of lncRNAs identified by RNA-Seq in tumor biopsy samples, normal tissues, and cell lines has shown a continuous and steep increase, with 15,941 lncRNA genes (28,031 transcripts) referenced in GENCODE (version 24 [7]), in comparison to 19,815 protein-coding genes, and more than 50,000 lncRNA genes reported by Iyer et al. [8]. These lncRNAs are associated with multiple biological processes such as development, cell differentiation or pathologies [9–11]. However, reliable and comprehensive genomic annotations of lncRNAs are not available for many species, such as livestock or crop species.

In this context, it is important to annotate this major fraction of the transcriptome in livestock species, for which several loci involved in complex and economically relevant traits [i.e. quantitative trait loci (QTL)] have been described but with limited success regarding the identification of the underlying causative mutation(s). Given that approximately 80% of the variants associated with human complex traits map outside of protein-coding exons of which 40% are in intergenic regions [12, 13], identifying the lncRNA repertoire is crucial to better understand the “genotype to phenotype” relationships in livestock [14, 15]. To date, few lncRNA studies have been reported for livestock species, apart from lncRNA studies in bovine [16] and trout [17], and the construction of multi-species databases such as NONCODE [18, 19] and the domestic-animal lncRNA database (ALDB) [20, 21]. Research programs are in progress on several farm species, e.g., in projects conducted within the framework of the Functional Annotation of Animal Genomes initiative [14, 15].

Different methodologies have been described to discover and model lncRNAs. This generates some variability in the number of putative lncRNAs reported and stresses the importance of precisely defining the tools and thresholds for each analysis step. Regarding lncRNA modeling, the FEELnc program (FlExible Extraction of Long noncoding RNAs), developed by Wucher et al. [22, 23], distinguishes lncRNAs from mRNAs based on a machine-learning method that estimates a protein-coding score according to different criteria such as the RNA size, ORF coverage and multi k-mer usage. One main advantage of the FEELnc program is its ability to derive an automatically computed cut-off that maximizes the lncRNA prediction sensitivity and specificity. In addition, and contrary to other tools such as CPC [24] or CPAT [25], FEELnc provides a lncRNA classification based on their genomic position with respect to a pre-defined set of reference genes (usually protein-coding genes), which allows to distinguish intergenic from intragenic lncRNAs and to sub-classify them according to their orientation with respect to a reference set of genes. Such a classification can be useful to formulate hypotheses about co-expression patterns observed between lncRNAs and their closest protein-coding genes.

In this context, our aim was to describe the chicken lncRNA repertoire. We focused on the liver and abdominal adipose tissues because of their importance in various economical traits for which energy storage and mobilization play key roles. The liver is a key organ for energy and lipid metabolism and homeostasis, and the adipose tissue plays a key role in lipid storage and mobilization when the organism is stressed or in transition phases. These two organs, through the regulation of the lipid metabolism (synthesis, storage and catabolism), are important for the bird’s adaptation to environmental changes [26–28]. Furthermore, both tissues are relatively homogeneous in cell composition. Both tissues were deeply sequenced (with an average of 100 million stranded paired-end reads per sample, totaling 1.65 billion per tissue) to capture weakly expressed lncRNAs and across a large number of biological replicates (16 birds per tissue) to obtain sufficient statistical power to assess correlations of expression levels between lncRNAs and their closest protein-coding RNAs.

In coordination with the FAANG initiative (FAANG Bioinformatics and Data Analysis subcommittee), we used a pipeline based on STAR, Cufflinks and FEELnc to describe and characterize a catalogue of expressed putative lncRNAs. We used two protein-coding score cut-offs (including a stringent one for lncRNAs) to partition our transcript set into lncRNAs, protein-coding RNAs and ambiguous RNAs (i.e., with intermediate protein-coding scores). We found approximately 2193 lncRNA genes (2979 transcripts), from which we extracted a reliable subset of 1670 genes (2412 transcripts) that were characterized by reproducible expression across the 16 replicates. We then compared their structure and expression levels to those of mouse and human lncRNAs. Using the FEELnc classification, we found interesting cases of co-expression between lncRNAs and their closest coding mRNAs, especially for pairs in divergent or antisense orientations. Overall, we provide a powerful and deeply characterized resource for investigating lncRNA relevance in the chicken liver and adipose tissue.

## Results and discussion

### Chicken lncRNAs predicted by FEELnc and their structure and expression features

For the liver and adipose tissue samples (16 replicates per tissue), we obtained on average 100 million stranded, paired-end reads. We compared the efficiencies of the recently published Stringtie and the classical Cufflinks programs to predict transcripts from our sequencing data, providing the Ensembl annotation as a guide and starting from the same BAM files generated by STAR. The Cufflinks/Cuffmerge pipeline processed our dataset of 32 samples in approximately 79 h and generated 39,504 transcripts for 22,413 genes. Stringtie took less than 3 h but produced approximately 4 times more predictions (150,659 transcripts for 108,098 genes), which included a majority of mono-exonic models (68 vs. 11% for Cufflinks). The number and the structure of the transcript models found with Stringtie in our data were considerably larger than expected based on data from the literature [6]. Thus, for this study, we used the more realistic models from Cufflinks/Cuffmerge. Finally, the STAR/Cufflinks/Cuffmerge pipeline applied to our 32 samples resulted in a more than two-fold increase in number of transcripts compared to that reported in the Ensembl V84.4 annotation on the reference GalGal4 genome, with 39,504 transcripts for 22,413 genes compared to the 17,954 transcripts for 15,508 genes in the Ensembl annotation.

**Fig. 1:**
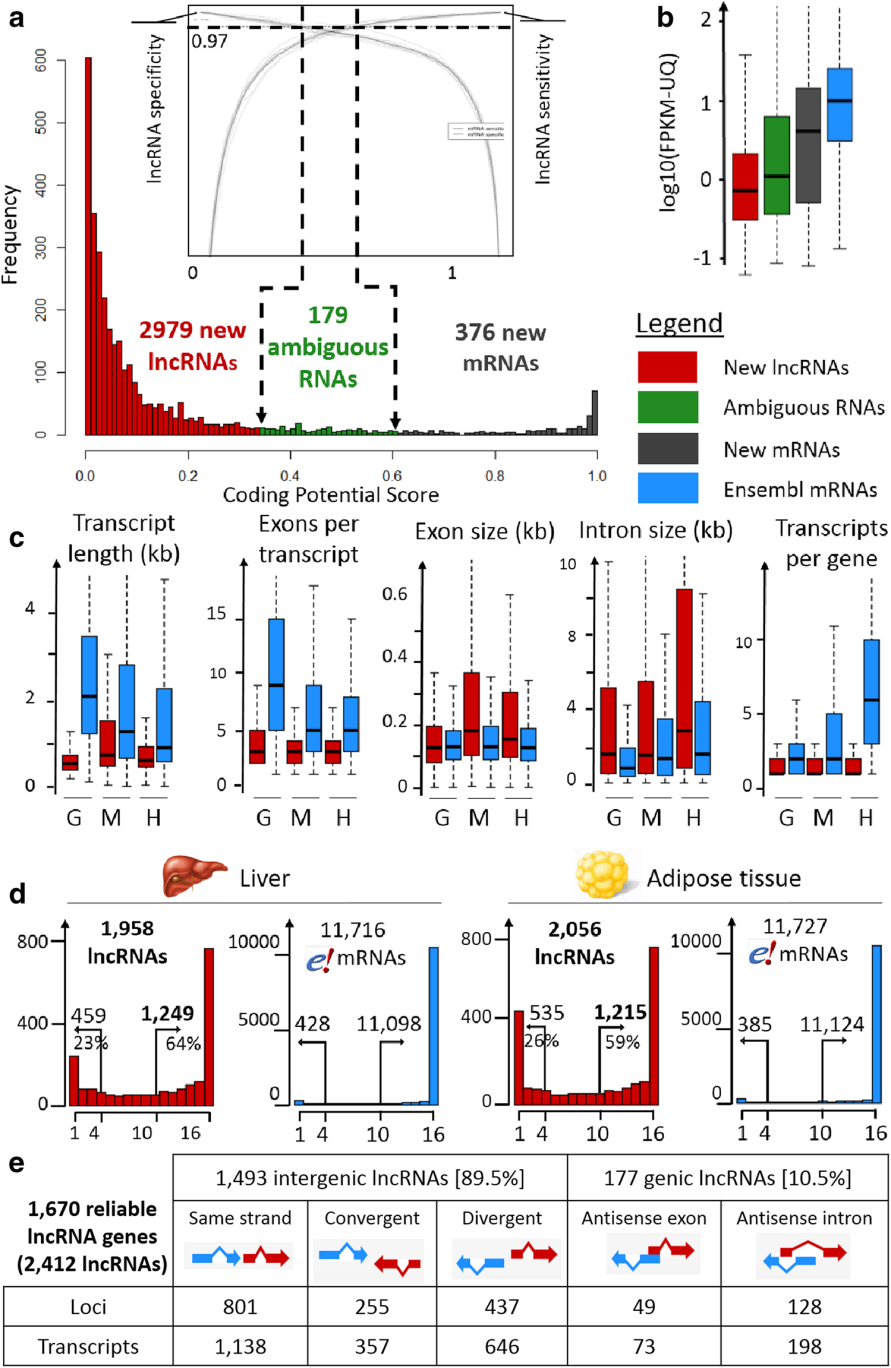
Predicted lncRNA features. **a** LncRNA prediction with a user-defined lncRNA specificity/sensitivity cut-off according to the two ROC curve graph provided by FEELnc. **b** Expression distribution within the three classes (new lncRNAs, ambiguous RNAs and new mRNAs) compared to that of known protein-coding genes from Ensembl. **c** Structural features for lncRNAs and Ensembl protein-coding RNAs in three species (G = *Gallus gallus*, M = *Mus musculus*, H = *Homo sapiens*). For the chicken lncRNAs, the data were generated in this study, while for the human and mouse lncRNAs, the data are taken from Ensembl V83. **d** Number of genes considered as expressed (FPKM-UQ ≥ 0.1) (*y*-axis) according to the number of biological replicates (*x*-axis) in the liver (*left*) and adipose tissue (*right*) for lncRNAs and Ensembl protein-coding genes. On each plot are indicated the number of genes for which at least 10 samples have a FPKM-UQ ≥ 0.1 (*right number*) and the number of genes for which a maximum of four samples have a FPKM-UQ ≥ 0.1 (*left number*). **e** Classification by FEELnc of the 1670 reliable lncRNA genes for 2412 transcripts

To date, no lncRNA has been annotated in the V84.4 Ensembl chicken gene dataset. These 39,504 newly modeled transcripts were then submitted to the “FlExible Extraction of Long noncoding RNAs” (FEELnc) pipeline to identify putative lncRNAs (see the “Methods” section). Fixing a specificity cut-off at 0.97 and using the NONCODEV5 database as the noncoding transcript training set (see the “Methods” section), we identified 2979 putative lncRNA transcripts (for 2193 genes), 376 new mRNAs (for 279 genes), and 179 ambiguous RNAs (Fig. 1a). When the training set of intergenic regions was used as the noncoding transcript training set (see the “Methods” section), we found 2588 lncRNA transcripts, with most of them (2539 lncRNAs) being common to the two final sets. Such a result shows the usefulness of FEELnc to predict lncRNAs in a species for which no lncRNA repertoire is available for training. We then compared our lncRNA set with the chicken lncRNAs available in the NONCODE and ALDB multispecies databases. We found that 14 and 25% of our chicken set was in common with the chicken NONCODE and ALDB datasets, respectively, using stringent criteria and 16 and 27% using more relaxed criteria (see “Methods” section). Note that the ALDB dataset shares 25% of the chicken lncRNAs with NONCODEV5 under the relaxed criteria. Such results highlight that lncRNA annotation strongly relies on the bioinformatics pipelines used for the gene modeling and lncRNA prediction but also on the RNA-Seq samples used in terms of sequencing depth, tissue analyzed and probably physiological status of the animals.

To evaluate the relevance of our chicken lncRNA set, we analyzed the gene expression profiles of the three classes “putative lncRNA transcripts”, “new mRNAs” and “ambiguous RNAs” and also compared the structural features of our lncRNAs with those of the mouse and human lncRNAs. As expected, the 2193 putative lncRNA genes are on average tenfold less expressed than the known or new protein-coding genes, and the ambiguous RNAs have an intermediate expression (Fig. 1b). This is in accordance with previous findings in mammals that showed that lncRNAs are far less expressed than protein-coding genes [6, 29–31]. Then, we characterized the structural features of these chicken putative lncRNA transcripts in comparison to the human and mouse lncRNAs available in Ensembl and compared them with the protein-coding RNAs available in Ensembl for these three species. Overall, the features observed for the chicken lncRNAs are consistent with those observed in mammals in the human and mouse ENCODE projects [6] (Fig. 1c). First, regardless of the species analyzed, lncRNAs are spliced but with fewer exons than the protein-coding RNAs, with medians of 3 and at least 5, respectively. Second, the median exon length is similar for lncRNAs and protein-coding RNAs in chickens (127 ± 1 nt). This is similar to what was found in humans and mouse, even if the chicken lncRNA exons are slightly longer than the protein-coding exons (for example, medians of 155 nt vs. 126 nt in humans, Wilcoxon–Mann–Whitney test, p value <2.2 × 10^−16^). Third, the lncRNA transcripts are shorter than the protein-coding transcripts in the chicken, as in humans and mouse, because of the observed smaller number of exons. In the chicken, the median transcript length is 529 nt for lncRNAs, compared to 2067 nt for protein-coding RNAs (Wilcoxon–Mann–Whitney test, p value <2.2 × 10^−16^). Finally, we observed a smaller number of isoforms per lncRNA gene in the three species compared to that of the protein-coding RNA genes, which was expected given that lncRNAs have a smaller number of exons [6].

In terms of the expression measured at the locus level (see the “Methods” section), the 2193 chicken lncRNA genes are characterized by at least one read in at least one replicate of one tissue (with 1958 in the liver and 2056 in the adipose tissue). To obtain a more reliable set of expressed lncRNAs, we took advantage of the large number of replicates to remove genes with low signals. Rau et al. [32] developed an R package (HTSfilter) for RNA-Seq data analysis to correctly filter out lowly-expressed genes and thereby increase the power of detection in the context of the differential expression of protein-coding genes. Unfortunately, this data-driven method (based on the Jaccard similarity index to calculate a filtering threshold) is not appropriate for lncRNAs because of their low expression level (see Additional file 1: Fig. S1). Therefore, we analyzed the reproducibility of the expression level across the 16 replicates of each tissue using the standard 0.1 FPKM-UQ threshold (see the “Methods” section). Figure 1d provides the numbers of long noncoding and protein-coding genes expressed according to the number of biological replicates for each tissue. Long noncoding genes show quite good reproducibility of expression across samples, with 1249 of them having an FPKM-UQ higher than 0.1 in at least 10 of the 16 samples in the liver, i.e., 64% of all hepatic lncRNA genes with one read in one sample (Fig. 1d, left). Note that 459 of the long noncoding genes (23%) have a poorly reproducible expression, with no more than four samples with an expression level higher than the threshold in the liver. Similar results were obtained for the adipose tissue (Fig. 1d, right), with 1215 lncRNA genes having an FPKM-UQ higher than 0.1 in at least 10 of the 16 samples. Combining these two sets of expressed lncRNAs results in 1670 genes. Finally, the further analyses were performed with these 1670 reliable long noncoding genes (for 2412 transcripts) that were robustly expressed in the liver and/or adipose tissue. These numbers of long noncoding genes are consistent with other studies that focus on a single tissue, even if the number of replicates, the sequencing depth and the criteria used to consider that a long noncoding gene is expressed, differ between studies. For example, Wang et al. [33] reported 2805 lncRNA transcripts in the pig endometrium (using 12 porcine samples and 85–105 million reads per sample), and Billerey et al. [34] reported approximately 1300 lncRNA transcripts in bovine muscle (using nine samples with 15 million to 45 million reads per sample). In contrast, multi-tissue studies reported a larger number of lncRNA transcripts, generally above 10,000, with a wide variation depending on the sequenced tissues and the tools used for the lncRNA detection (9778 lncRNA transcripts reported by Koufariotis et al. [16] in 18 bovine tissues (using 1.87 million 120-bp stranded paired-end reads and CPC/CNCI tools for lncRNA prediction [24, 35]), and 20,163 lncRNA transcripts reported by Li et al. [36] in 13 maize tissues (using 1.17 million 35- to 110-bp unstranded paired- and single-end reads and the CPC tool for lncRNA prediction [24]).

Using the FEELnc classifier module, we then analyzed the class distribution of the 1670 reliable FEELnc lncRNA genes compared to annotated protein-coding genes from Ensembl (Fig. 1e). We found 1493 intergenic lncRNA genes (89%), which was the largest class as reported in humans by Derrien et al. [6], compared to 177 intragenic lncRNA genes (11%). These 1670 lncRNA genes, which are characterized by a good reproducibility of expression level in at least one of the two tissues and corresponding to 2412 transcripts, were analyzed more deeply and are reported in Additional file 2: Table S1.

### Distribution of LncRNAs across chicken macro- and micro-chromosomes

**Fig. 2:**
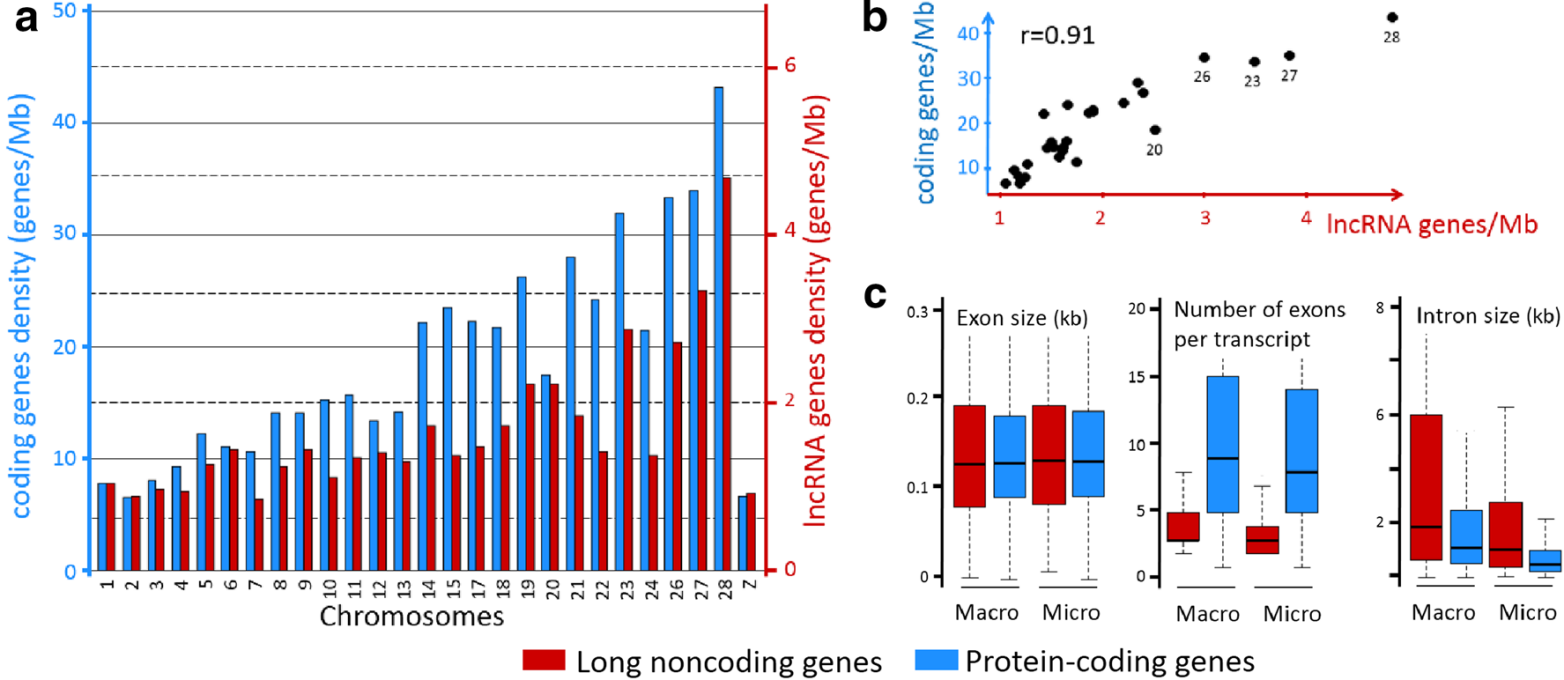
Gene density and structural features for protein-coding genes and lncRNA genes across the chicken macro- and micro-chromosomes. **a** Gene density for all chromosomes (except for chromosomes 16, 25, and W that are not sufficiently well sequenced). **b** Correlation of gene densities between protein-coding genes (*y*-axis) and long noncoding genes (*x*-axis). **c** Exon size, exon number and intron size for macro-chromosomes 1–5 and micro-chromosomes 20, 21, 23, 26, 27 and 28

Because the chicken genome, similar to most avian genomes, has chromosomes of markedly different lengths (termed macro- and micro-chromosomes), the genomic distribution of putative lncRNA transcripts was investigated. This analysis was restricted to chromosomes with nearly complete sequence coverage, which excluded chromosomes *Gallus gallus* GGA16 and 25 [37]. For lncRNAs, we found a negative correlation between gene density and chromosome length, as previously reported for protein-coding genes [37] (Fig. 2a, b). Both macro- and micro-chromosomes are known to have properties such as a high G+C content, recombination rate and gene density [37]. Moreover, in [37] a strong correlation was observed between the length of a gene and the size of the chromosome, mostly due to variations in intron size. Therefore, we analyzed the intron and exon lengths between macro- and micro-chromosomes for lncRNA and protein-coding genes (Fig. 2c). Although exon lengths do not vary significantly between both chromosome types, intron lengths are greater for macro-chromosomes than for micro-chromosomes, which explains the higher gene density on micro-chromosomes; these observations were similar for protein-coding and long noncoding RNAs.

### Conservation of lncRNAs between chicken and human genomes

**Fig. 3:**
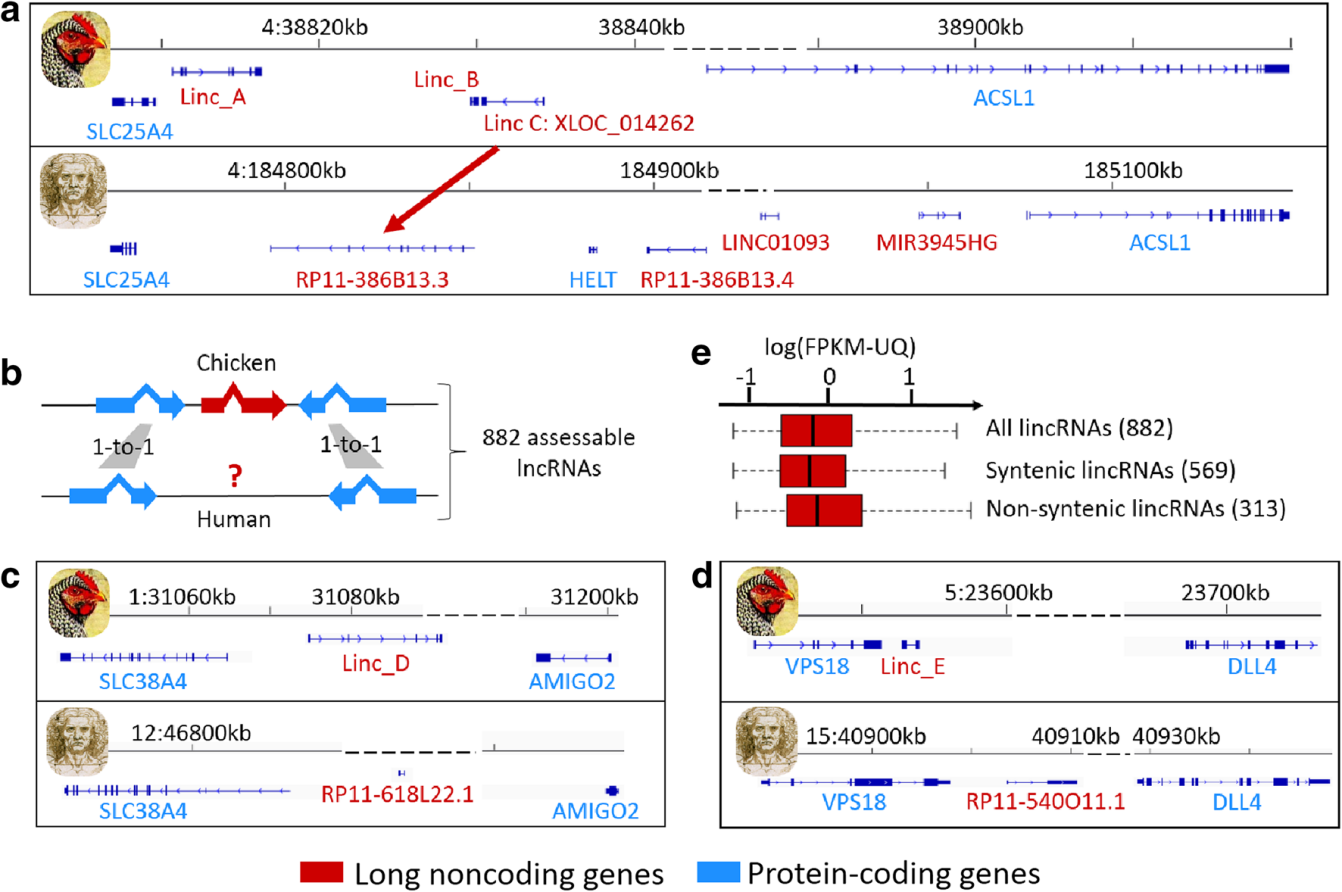
Chicken/human lncRNA conservation in terms of sequence (**a**) and syntenic position (**b**–**e**). **a** An example of chicken lncRNA (*XLOC_014262*) that has a conserved sequence with the human *RP11*-*386B13.3* lncRNA and a similar syntenic position in both species. **b** Schematic picture illustrating our approach for identifying syntenic lncRNAs between the chicken and human genomes. **c**, **d** Schematic representations of the *SLC38A4*-*AMIGO2* (**d**) and *VPS18*-*DLL4* loci. **e** Distributions of the expression of the two subsets of lncRNAs with conserved or not synteny

We evaluated the degree of sequence similarity between chicken and human lncRNA transcripts by performing all pairwise sequence comparisons. Even by using relaxed criteria (see the “Methods” section), we found no match for our lncRNA set except for two transcripts *XLOC_006973* (360nt) and *XLOC_014262* (445 nt), for which more than 60% of the chicken lncRNA sequences matched with 26% of the two associated human lncRNA sequences, *RP11*-*20B24.2* (895 nt with 72% identity) and *RP11*-*386B13.3* (1192 nt with 94% identity), respectively. These results are consistent with previous studies that reported that the number of lncRNAs with sequence conservation decreases as the phylogenetic distance increases [6, 31, 38]. Note that the second lncRNA, *XLOC_014262*, which has a conserved sequence with the human *RP11*-*386B13.3* lncRNA, also displays synteny conservation between the chicken and human genomes (see Fig. 3a). Such sequence and synteny conservations between these two species that diverged approximately 300 Myr ago suggest an important functional role of this lncRNA. Moreover, XLOC_014262 is highly expressed in the liver (FPKM-UQ = 0.43 on average), in contrast to the adipose tissue (FPKM-UQ = 0.06 on average), and is located at 21 kb from the neighboring protein-coding gene *SLC25A4* (that encodes a protein involved in the exchange of cytoplasmic ADP with mitochondrial ATP across the mitochondrial inner membrane). Interestingly, *XLOC_014262* and *SLC25A4* are significantly and positively co-expressed in the liver (r = 0.64, p value = 0.013). Taken together, these results suggest a regulatory role of this lncRNA in the liver metabolism, and maybe in energy metabolism. Complementary to this first analysis, we further analyzed synteny conservation of lncRNAs between the chicken and human genomes. In our approach (see Fig. 3b), we only considered long intergenic noncoding RNA genes (lincRNAs) that were surrounded by two protein-coding genes that had a 1-to-1 orthologous relationship with the human genome (Ensembl v.83). For these 882 lncRNA genes, we then considered that there was synteny conservation for a lncRNA gene if a human lncRNA gene was located between the two orthologous protein-coding genes, with the same configuration of the trio in terms of order and orientation. We found that 64% (569) of our lncRNA genes met this criterion. Two examples of lncRNAs with synteny conservation are provided in Fig. 3c for the *SLC38A4*-*AMIGO2* locus and in Fig. 3d for the *VPS18*-*DLL4* locus. Previous studies have shown similar results: Ulitsky et al. [39] reported intergenic lncRNAs in conserved positions in the zebrafish, human and mouse genomes without detectable sequence conservation. The same team analyzed this phenomenon more deeply using various phylogenetically distant species [38] (mammals, chicken, lizard, coelacanth, sea urchin, etc.) and confirmed that a large fraction of the lncRNAs that displayed synteny conservation were highly divergent at the nucleotide level. The same observation was recently reported in plants between *Brassecaceae* and *Cleomaceae* [40]. Regarding the proportion (36%) of lncRNA genes that did not display synteny conservation, different hypotheses can be drawn. First, this gene subset does not have specific properties in terms of expression level (Fig. 3e) or structural features (data not shown) compared to the gene subset that displays synteny conservation, which allows us to discard such features for explaining these two non-syntenic versus syntenic lncRNA subsets. Even if the human genome annotation is more advanced than that of the chicken genome, a first hypothesis is that the human genome annotation is not complete in terms of lncRNAs, as suggested by recent studies that have enriched the list of lncRNAs [8]. Another hypothesis is that lncRNAs are more species-specific than protein-coding genes because of their major roles in the regulation of gene expression. This hypothesis is supported by a higher rate of synteny conservation for protein-coding genes than for lncRNA genes. Indeed, the “syntenic conservation” analysis performed for lncRNAs was also performed for the protein-coding genes found between two orthologous protein-coding genes, and we observed that only 10% of the protein-coding genes do not display synteny conservation between the chicken and human genomes, compared to 36% for lncRNA genes.

### LncRNAs are less expressed and more tissue-specific than mRNAs in the liver and adipose tissues

**Fig. 4:**
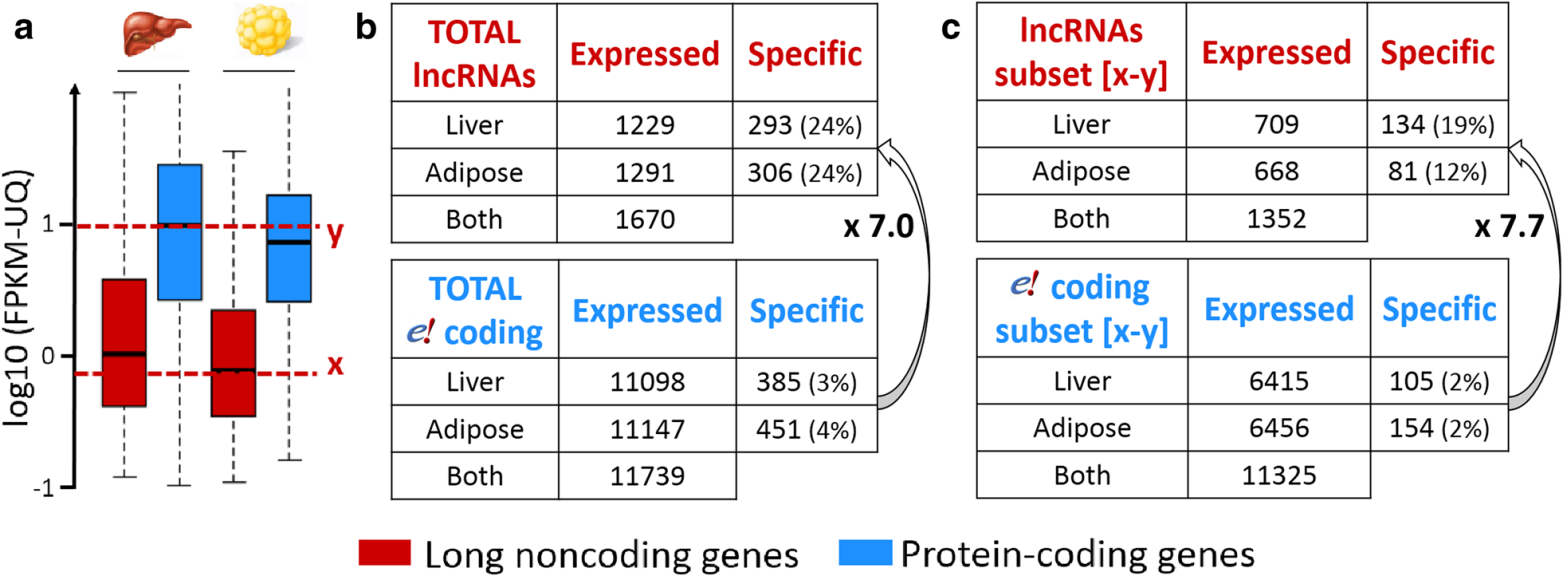
Tissue expression of lncRNA and protein-coding RNA genes in liver and adipose tissue in chicken. **a** Expression levels in both tissues. **b** Tissue-specific expression for the whole lncRNAs and Ensembl protein-coding RNAs. **c** Tissue-specific expression for a subset of the lncRNAs and protein-coding RNAs with similar expression (between the extreme medians of the lncRNA and mRNA expression distributions represented by x = 0.76 and y = 9.94 FPKM-UQ, respectively). The read counts were normalized for library size and gene size, and the biological replicates per tissue were taken into account as explained in “Methods” section

The patterns of expression of the lncRNA and mRNA genes clearly show that the lncRNAs are less expressed than the protein-coding genes in both tissues (Fig. 4a). The median FPKM-UQ for lncRNAs (approximately 1) is tenfold lower than that for protein-coding RNAs (approximately 10), and the third quartile of the lncRNA gene expression is close to the first quartile of the protein-coding gene expression. This lower expression level is consistent with previous studies in other organisms [6, 29]. We analyzed the degree of tissue specificity for both gene types (Fig. 4b, c). Because the lncRNAs are weakly expressed with numerous genes having an FPKM-UQ higher than 0.1 in only a few of the 16 samples of a tissue (see Fig. 1d), we defined a gene that was expressed in one tissue (i.e., with a FPKM-UQ higher than 0.1 for at least 10 of the 16 samples) as non-expressed in the second tissue if its FPKM-UQ was lower than 0.1 in more than 12 samples (see the “Methods” section and Fig. 1d). Based on this definition, on average 24% of the lncRNAs are specifically expressed in one tissue, compared to only 3.5% for protein-coding genes (on average a sevenfold difference, Fisher test, p value <2.2 × 10^−16^) (Fig. 4b). These differences between lncRNAs and protein-coding genes are not due to the lower expression levels of lncRNAs because we also found similar differences between lncRNAs and protein-coding genes that are expressed at similar levels (Fig. 4c). These differences remain significant and similar when we used either more stringent or more relaxed criteria across replicates to determine expression in one tissue or no expression in the second tissue. For example, we found a 9.5-fold difference with stringent criteria (16 of the 16 replicates with a FPKM-UQ higher than 0.1 in one tissue and no sample with a FPKM-UQ higher than 0.1 in the second tissue) and a 5.9-fold difference with more relaxed criteria (at least 8 of the 16 replicates with a FPKM-UQ higher than 0.1 in one tissue and no more than 8 samples with a FPKM-UQ higher 0.1 in the second tissue). Although we analyzed tissue specificity between only two tissues, these results are consistent with previous reports in other organisms that analyzed lncRNAs in several tissues, as in Cabili et al. [31] on 24 tissues and cell types or in Derrien et al. [6] on 16 tissues.

To evaluate the relevance of these tissue-specificity gene sets, we performed a GO term enrichment analysis for the protein-coding gene subsets with DAVID [41, 42] (see Additional file 3: Table S2). As expected, for the liver-specific protein-coding gene subset, we found an enriched GO term cluster related to lipid metabolism that was supported by well-known liver-specific genes such as those coding for hepatocyte nuclear factors (HNF1A, HNF4, NR1H4), apolipoproteins (APOB, APOA4) or enzymes involved in cholesterol catabolism and bile acid metabolism (CYP7a1, HSD3B7, SLCO1A2). For the adipose-specific protein-coding gene subset, an enriched GO term cluster related to development and morphogenesis was identified, which was supported in particular by several *HOX* genes involved in body fat mass control and obesity [43, 44]. This cluster of genes is likely related to the capacity of white adipose tissue to expand and differentiate. The four subsets of adipose- and liver-specific genes for long noncoding and protein-coding genes are in Additional file 4: Table S3.

### Co-expression of LncRNAs and their nearest protein-coding genes

Long noncoding RNAs are emerging as new players in multiple mechanisms of cell machinery, including regulation of gene expression. Even if they can act over long distances to activate transcription at distal promoters [45], it has been demonstrated that they can also locally affect the gene expression of their neighboring protein-coding genes [11, 30, 46]. Concerning these “local” regulations leading to co-expression, we can distinguish genic lncRNAs that overlap protein-coding genes in an anti-sense orientation from intergenic lncRNAs in a divergent orientation with respect to their closest protein-coding genes. These latter lncRNAs may share a common bidirectional promoter with their closest protein-coding genes if the distance between them is less than a certain threshold, often fixed at 1 kb [47–49]. Hence, we evaluated the co-expression of each “lncRNA—nearest protein-coding RNA” pair across all the samples of each tissue according to two criteria: (1) the FEELnc classification, and (2) for the three intergenic lncRNA classes, a distance of less than 1 kb between the two genes considered. For some classes, we expected a larger number of significantly co-expressed pairs when the genes of a pair are closer together than when they are further apart, based on the hypothesis that a lncRNA is more likely to contribute to the regulation of a protein-coding gene if it is close to it.

We observed that pairs of divergent lncRNA genes and close (≤1 kb) to protein-coding genes are more significantly co-expressed than the more distant divergent pairs (22 vs. 13%, respectively, Fisher test, p value <0.05) (Table 1). Similar results were found for divergent protein-coding gene pairs (30 vs. 22%, Fisher test, p value <0.1) (Table 1). These results suggest that very close divergent pairs of lncRNAs and protein-coding genes are controlled by the same promoter. The same observation was previously reported for lncRNA-coding RNA pairs that were referenced by Schmitz et al. [11], as well as for protein-coding RNA pairs [47–49]. Interestingly, we showed that most of the correlated gene pairs are positively correlated: this occurs in more than 82% of cases, regardless of the type of gene pair (mRNA–mRNA or lncRNA-mRNA). Such a result is consistent with other studies conducted in mammals for mRNA–mRNA pairs [48] and supports the hypothesis that most bidirectional promoters positively regulate the co-expression of gene pairs, whereas a minority of bidirectional promoters induce the transcription of one gene while inhibiting the transcription in the other direction. The detailed mechanisms that underlie the co-expression of divergent RNA pairs can be multiple and involve cis- or trans-regulatory elements [11, 47, 50].

**Table 1:** Significant correlations between expression for lncRNA-mRNA and mRNA–mRNA pairs considering FEELnc classes and distance between genes

	Same strand	Convergent	Divergent	Antisense exon	Antisense intron
Genes	674	194	370	23	100
lncRNA-mRNA					
≤1 kb	51/91 (56%) +49/−2	5/28 (18%) +3/−2	23/105 (22%) +19/−4	5/23 (22%)	13/100 (13%)
>1 kb	139/583 (24%) +127/−12	13/166 (8%) +10/−3	34/265 (13%) +27/−7		
p value	2.37 × 10^−9^	NS (0.15)	3.7 × 10^−2^	NA	
mRNA–mRNA					
≤1 kb	28%	17%	30%	24%	
>1 kb	22%	19%	22%		
p value	NS (0.19)	NS (0.80)	0.09	NA	

The FEELnc classes are “same strand”, “divergent” and “convergent” for intergenic lncRNAs, and “antisense exon” and “antisense intron” for genic lncRNAs

For intergenic lncRNAs, co-expression was tested according to the physical distance (1 kb) between the two genes considered

The difference in correlated pairs between the “distance” sets was tested by a Fisher test (NS: non-significant). Note that this co-expression table depends on the modeling of protein-coding genes obtained by Ensembl V84.4

Regarding genic lncRNA-mRNA pairs, lncRNAs oriented in the antisense direction with respect to an exon or intron of a protein-coding gene are significantly co-expressed (22 and 13%, respectively) with the overlapping protein-coding gene (Table 1). Several cases of co-expression of genic lncRNA-mRNA pairs in an antisense orientation have been reported, and the modes of action of such lncRNAs on the regulation of mRNA loci are multiple and complex [11, 51–54]. Strikingly, we found that the significant correlations between lncRNA and mRNA levels are positive. Derrien et al. [6] also reported a majority of positive co-expressions for lncRNA-mRNA pairs in an anti-sense orientation. The mechanisms that underlie such positive co-expression seem to be complex and act at distinct regulatory levels including the translation, splicing and transcription levels [55–58].

In the same strand pair category, lncRNAs are more significantly correlated with their proximal protein-coding neighbors (≤1 kb) than with distant RNAs (56 vs. 24%, respectively) (Table 1). Most of these lncRNA genes probably have to be considered as an extension of the protein-coding gene, which implies that the Cufflinks/Cuffmerge procedure could not model full-length lncRNAs. Indeed, such a difference is not observed for the protein-coding gene pairs, considered as better characterized and used here as a control (28 and 22% for the two distance subsets) (Table 1).

Next, we focused on two lncRNA-mRNA pairs that were significantly correlated in the liver, i.e. one divergent pair and one exon antisense pair.

### Specific cases of divergent and exonic antisense lncRNA-mRNA pairs that are significantly correlated in liver

Our aim was to identify pairs with a protein-coding gene involved in lipid metabolism, to be able to hypothesize a regulatory role of the lncRNA on its neighboring coding gene [59]. Three long noncoding genes were previously described in mammals as being involved in lipid homeostasis: the liver-enriched *lncLSTR*, reported as a putative regulator of the plasma triglyceride level in mice [60]; the lncRNA *HULC*, which is abnormally expressed in hepatocellular carcinoma cells and has been shown to increase the triglyceride and cholesterol levels in these cells [61]; and the antisense lncRNA *APOA1*-*AS*, which was shown in humans and monkeys to negatively regulate *APOA1* expression (a major component of high-density lipoprotein) [62]. Surprisingly, these long noncoding genes, absent from the Ensembl chicken V84 annotation, were not modeled with our RNA-Seq data, and a manual inspection using the Integrative Genomics Viewer confirmed that no reads were mapped at the putative genomic locus, contrary to the neighboring protein-coding genes (see Additional file 5: Figure S2). These results suggest that these long noncoding genes are either absent in the chicken genome or not systematically expressed in the liver, regardless of the age, sex and physiological state of the individuals.

For the set of antisense lncRNA-mRNA pairs, no mRNA was found to be clearly involved in lipid metabolism according to the literature. Therefore, we analyzed the co-expression of one pair related to the protein-coding gene, *NPNT*, which was recently shown to play a role in the liver [63]. For the set of divergent lncRNA-mRNA pairs, we focused on a lncRNA related to the *DHCR24* gene known to encode a key enzyme of the biosynthesis of cholesterol, which has not been reported so far.

#### Exonic antisense lncNPNT-AS and NPNT protein-coding gene

**Fig. 5:**
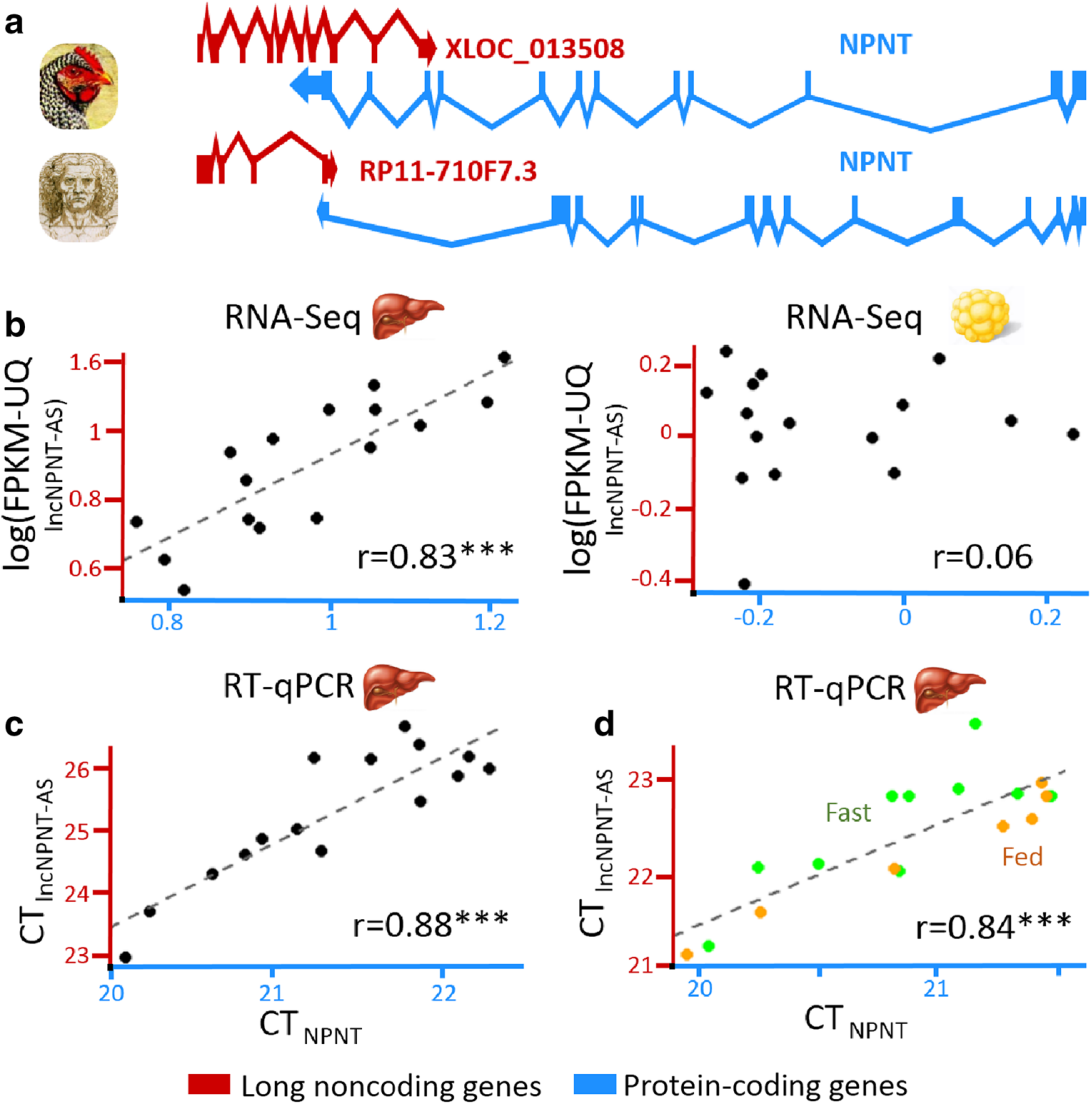
*NPNT* gene and its antisense lncRNA gene. **a** Gene models of the lncRNA/mRNA pair in the chicken and human genomes. **b** Expression of the lncRNA/mRNA pair analyzed with RNA-Seq data in liver (*left*) and adipose tissue (*right*). **c** Expression analysis with RT-qPCR data. **d** Expression of 20 fed and fasted birds (analyzed by RT-qPCR). Correlation significance: ***p value <0.001

As shown in Fig. 5, the *NPNT* locus has the same gene organization in the chicken and human genomes, with a lncRNA (called *RP11*-*710F7.3* in the human genome) that overlaps the *NPNT* protein-coding gene in an antisense orientation (Fig. 5a). Nevertheless, the intron–exon structure of these two genes and the exonic region of the *NPNT* that overlaps the lncRNA differ in the two species. The highly significant correlation found by RNA-Seq between the two chicken *lncNPNT*-*AS* and *NPNT* genes in the liver (Fig. 5b, left) was fully validated by RT-qPCR experiments (Fig. 5c). We also found a positive correlation between the hepatic expression of the two genes in other chickens with fed and fasted statuses (Fig. 5d). Conversely, no significant correlation was observed in the adipose tissue (Fig. 5b, right). The *NPNT* gene encodes nephronectin, which is an extracellular matrix protein known to play a critical role in kidney development. However, its physiological role in the liver remains unclear. A recent study showed that *NPNT* expression is up-regulated in mouse and human hepatitis [63]. Our results suggest a positive regulatory role of the antisense *lncNPNT*-*AS* on *NPNT* expression, but the regulatory mechanisms that underlie this positive co-expression and its functional impact in the liver remain to be elucidated.

#### DHCR24 and its divergent lncRNA

**Fig. 6:**
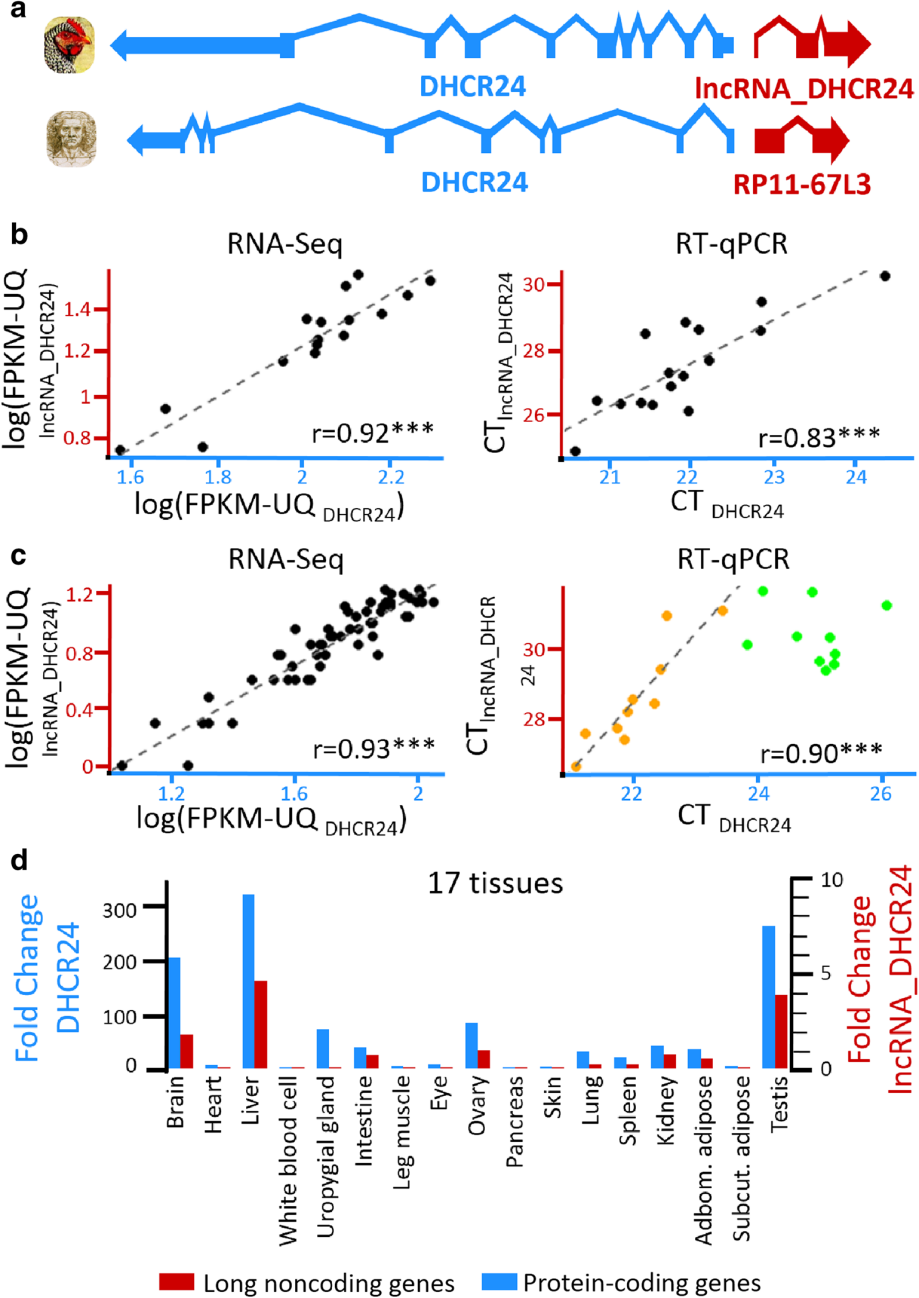
*DHCR24* gene and its divergent lncRNA gene. **a** Gene models of the lncRNA/mRNA pair in the chicken and human genomes. **b** Expression correlation in liver using RNA-Seq data (*left*) and confirmed by RT-qPCR (*right*). **c** Expression in adult birds analyzed by RNA-Seq (*left*) and young birds under fasted and fed statuses analyzed by RT-qPCR (*right*). **d** Expression across 17 tissues (see the “Methods” section). Correlation significance: ***p value <0.001

We found the same gene pair organization at the *DHCR24* locus in the human and chicken genomes, with a lncRNA gene (called *RP11*-*67L3* in the human genome) that is transcribed in the opposite direction with respect to the *DHCR24* protein-coding gene (Fig. 6a). The significant correlation found by RNA-Seq between the two chicken *lncRNA_DHCR24* and *DHCR24* expression levels in the liver (Fig. 6b, left) was confirmed by RT-qPCR (Fig. 6b, right), with similar correlation coefficients. No significant correlation (r = 0.064, p value = 0.81) was observed for the adipose tissue, in which both genes are less expressed than in the liver (FPKM-UQ = 20.2 vs. 0.9, respectively, for *lncRNA_DHCR24*, and 112.7 vs. 17.6, respectively, for *DHCR24*). These two divergent gene pairs were positively co-expressed in various experimental designs, including young and adult chickens with fed and fasted statuses (Fig. 6c). The analysis of expression across 17 chicken tissues also showed a co-expression of the two *DHCR24* and *lncRNA_DHCR24* genes, with the highest RNA levels in the liver, brain, testis and ovary (Fig. 6d).

The tissue expression pattern is consistent with the physiological role of *DHCR24* since it encodes the last enzyme necessary for cholesterol synthesis, with cholesterol being the precursor of the biosynthesis of the steroid hormone. To our knowledge, such co-expression observed in different physiological conditions between *DHCR24* and a divergent lncRNA has never been reported before; it suggests that the two members of this gene pair that are in a divergent orientation and at a small distance between the transcription start sites (202 bp) share an active bidirectional promoter. Further experiments are required to determine if this promoter can initiate transcription in both directions. The strong co-expression that was observed in several experimental designs suggests a regulatory role of the *lncRNA_DHCR24* on *DHCR24* expression and thereby on the biosynthesis of cholesterol. Similar to *lncLSTR* [60] or *APOA1*-*AS* [62], *lncRNA_DHCR24* thus constitutes a novel candidate gene to be added to the list of lncRNAs involved in lipid metabolism regulation.

## Conclusions

Our study aimed at establishing a first repertoire of the lncRNAs in the chicken liver and adipose tissue, two tissues that are known to be important for energy homeostasis and lipid metabolism. We characterized this repertoire in terms of structure, expression and co-expression with respect to protein-coding genes, based on 16 biological replicates per tissue. In terms of structure, we observed a large subset of lncRNAs that were conserved by position between the chicken and human genomes but that were highly divergent at the nucleotide level. Although this latter observation was also reported in other studies [6, 17, 38, 64–66], complementary strategies could be considered for analyzing splice site sequence conservation [67]. Nevertheless, this reinforces the question regarding the functional meaning of syntenic conservation in the absence of sequence conservation, which does not rule out the conservation of the secondary structures of lncRNA sequences. More specific to the chicken genome, lncRNAs have the same chromosomal distribution as protein-coding genes in terms of gene density and length, with more and shorter genes on the micro-chromosomes. In terms of expression, the chicken lncRNAs are less expressed and more tissue-specific than the protein-coding genes, as previously reported for human and murine lncRNAs, supporting the important role that is attributed to lncRNAs as regulatory elements involved in tissue-specific functions. In terms of co-expression, 22% of the antisense overlapping lncRNA-mRNA pairs are significantly and positively co-expressed, thus providing new candidate genes to investigate the mechanisms that underlie such regulations. We show that divergent lncRNA genes are more significantly co-expressed with their close (≤1 kb) protein-coding genes than with more distant genes, suggesting the existence of active bidirectional promoters in the chicken. In particular, the *DRCH24* gene and its divergent lncRNA are highly co-expressed in various conditions in the liver, revealing a new lncRNA that might have an important role in the regulation of cholesterol synthesis.

## Methods

### Sample collection, RNA isolation and RNA sequencing

The liver and abdominal adipose tissue were extracted from 16 male chickens slaughtered at 9 weeks of age. Chickens were feed-deprived for 12 h and then fed again for 3 h before being euthanized by decapitation and bleeding. Immediately after slaughter, the liver and abdominal adipose tissue were removed, frozen in liquid nitrogen and then stored at −80 °C until the analyses.

Approximately 30 mg of liver and 100 mg of adipose tissue were homogenized in TRIzol reagent (Invitrogen, California, USA), and the total RNA was then extracted according to the manufacturer’s instructions, re-suspended in 50 µL of RNase-free water and stored at −80 °C. The total RNA was quantified with a NanoDrop^®^ ND-1000 spectrophotometer (Thermo Scientific, Illkirch, France). A260/280 and A260/230 ratios were greater than 1.7 in all samples, ensuring the purity of the preparation. The RNA quality was verified using an Agilent 2100 Bioanalyzer (Agilent Technologies France, Massy, France). The average RNA integrity numbers were 8.65 ± 0.47 (mean ± SD) for the two tissues: 9.4 ± 0.5 for the liver and 8 ± 0.6 for the abdominal adipose tissue.

Sequencing was conducted on 24 samples (16 livers and eight abdominal adipose tissue samples) and an additional eight abdominal adipose tissue samples, in a stranded and paired end manner with 2 × 100 bp, on a HiSeq 2000 (Illumina) and HiSeq 3000 (Illumina), respectively. Libraries with an on average 230-bp insert were prepared following Illumina’s instructions by purifying poly-A RNAs (TruSeq RNA Sample Prep kit). Illumina adapters containing indexing tags were added for subsequent identification of samples. Samples were PCR-amplified, and quantitative PCR was then performed for library quantification (QPCR NGS Library Quantification kit). All samples were filled on two to five lanes with a flow cell to minimize the inter-lane bias. After sequencing, the samples were de-multiplexed, and the indexed adapter sequences were trimmed using CASAVA v1.8.2 software (Illumina). We obtained 101 million reads per sample on average (111 million reads for the liver and 92 million reads for the adipose tissue), with a total of 3.3 billion reads for the 32 samples.

### Pre-processing steps on RNA-Seq data

Three billion reads from the RNA sequencing were mapped onto the chicken Galgal4 reference genome using STAR (v2.4.0i) [68], and the PCR duplicates were removed for each RNA-Seq sample using the SAMtools rmdup tool (v0.1.19) [69]. All the data were merged into one bam file with the merge tool (v1.1) from the Samtools suite to create the input file used to model transcripts and genes. Gene modeling was performed with both Stringtie (v1.0.1) [70] and Cufflinks (v2.2.1) [71], using the Ensembl gene annotation file (release 82) as a reference. To compare the results, tests were conducted under the same conditions with 12 cores. The CPU was an Intel(R) Xeon(R) CPU E5-2670 v2 @ 2.50 GHz. The counting step was performed by featureCounts (v1.4.5-p1) [72] with standard options but using both the multi- and the mono-mapped read options. Note that separated “.bam” files (one per sample) including the PCR duplicates were used for this counting step. We obtained 2.418 billion mapped reads with the ‘no multi-mapping’ option and 2.487 billion reads with the ‘multi-mapping’ option. Therefore, only 2.8% of the total reads were multi-mapped and these were discarded from further analyses. After completing all the filtering steps, we obtained an average number of mapped reads per sample of approximately 75 million overall (88 million and 63 million for the liver and adipose tissue, respectively). Each command line and input/output file used to run the different analyses are in Additional file 6.

### Long noncoding RNA prediction

lncRNA annotation was performed by the FEELnc program (FlExible Extraction of Long noncoding RNAs, v.23/11/2015 [22, 23]. Briefly, FEELnc is an alignment-free software that uses multi *k*-mer frequency data and relaxed open reading frame (ORF) annotation as the main computational features/predictors to discriminate protein-coding from non-coding RNAs. These features are then used in a machine-learning algorithm (random forest) to compute a coding potential score (CPS) that will discriminate between mRNAs and lncRNAs. In particular, the program can be self-trained with species-specific annotations and it automatically defines the coding potential threshold that maximizes the classification performance (i.e., where the sensitivity equals the specificity). Once the FEELnc model is trained with the above predictors, it is then applied on a set of novel transcript models (e.g., from Cufflinks or Stringtie) reconstructed after transcriptome sequencing to predict their protein-coding capacity. The description of the FEELnc program is accessible at bioarxiv [23] in which extensive benchmarking of the program in comparison with six other programs is presented based on the GENCODE human and mouse gold-standard datasets. Basically, FEELnc has three modules: “FEELnc_filter”, “FEELnc_codpot” and “FEELnc_classifier”. Using the first module “FEELnc_filter”, we filtered out all transcripts for which exons overlapped in the sense protein-coding exons or pseudogenes that are referenced in the chicken V78 Ensembl annotation. Note that the V78 Ensembl annotation is equivalent to the last V84.4 annotation for the chicken, with 15,508 coding genes and 17,954 coding transcripts. We also filtered out transcripts that were shorter than 200 bp according to the commonly accepted definition of long noncoding RNAs. The second module “FEELnc_codpot” separates putative long noncoding RNAs (lncRNAs) from protein-coding RNAs by first computing a coding potential core (CPS, ranging from 0 to 1) for each transcript and then computing a CPS cut-off that maximizes both the lncRNA sensitivity and specificity using a tenfold cross-validation according to the input training files. For the training set of protein-coding transcripts, we used the 15,508 known coding transcripts annotated by Ensembl. For the training set of long noncoding transcripts, we used both the 13,085 chicken putative transcripts from the NONCODEV5 database (v.2016) [18, 19] and a set of 11,000 genomic intergenic regions automatically extracted by FEELnc. Note that the lncRNA predictions of NONCODE are mainly based on the analysis of the Cufflinks gene models by the coding-non-coding index (CNCI) method [35]. Here, the CPS calculation is based on ORF coverage, mRNA size and multi k-mer frequencies; for this latter criterion, we chose frequencies of 1-, 2-, 3-, 6-, 9- and 12-mers, and the optimal performance in terms of specificity for our training data was 0.96. FEELnc allows the user to increase the performance metrics to obtain high-confidence predictions of lncRNAs/mRNAs, although this option leads to the creation of an intermediate category of ambiguous coding/noncoding transcripts (TUCp). The third module “FEELnc_classifier” classifies each lncRNA with respect to its location and orientation compared to its closest annotated protein-coding genes. The two main classes are (1) the genic lncRNA class, corresponding to lncRNA transcripts that overlap a protein-coding gene, and (2) the intergenic lncRNA class, with three subtypes that are the divergent, convergent and same-strand sub-classes, as detailed on the FEELnc website [22] and schematized in Fig. 1e. Each command line and input/output file used to run the different analyses are available in Additional file 6.

### Comparison of our lncRNA set with the chicken lncRNAs from the NONCODE and ALDB databases

The multi-species NONCODE [18, 19] and ALDB [20, 21] databases contain 9343 and 6132 chicken lncRNAs, respectively, that are either intergenic or overlap a gene in antisense orientation. The exon coordinates of our chicken lncRNA set were compared to those of both databases using the “bedtools intersect” tool v.2.25.0 [73]. Two thresholds were used i.e. 100% (stringent criteria) and 50% (relaxed criteria), which refer to the percentage of the lncRNA exon lengths in our dataset that match those of the analyzed database set. Because of the non-perfect modeling of lncRNAs, we considered that a lncRNA was present in two sets if at least one exon was shared by these sets.

### Sequence conservation

Sequences of human lncRNA transcripts were downloaded from the GRCh38 Ensembl database, version 83. Sequence comparisons between our chicken FEELnc sequences and the human sequences were conducted using the Blast software suite [74] (blastn V2.4.0+, with a word size of 7). The thresholds used for the FEELnc and human transcript comparison were 50% for the query coverage and 70% for the identity percentage.

### Syntenic conservation

A syntenic conservation analysis was performed for the lncRNA genes that were surrounded by two neighboring protein-coding genes with a 1-to-1 orthologous relationship with the human genome (Ensembl v.83, Biomart web-based tool [75, 76]). We considered that there was synteny conservation for a lncRNA if a lncRNA was also found in the human (GrCh37) between the same two coding genes, with the same orientation and the same order. Note that no upper limit was used in terms of distance between the lncRNA and the nearest protein-coding genes, but most of the distances are between 6 nt (min) and 35,000 nt (third quartile).

### Expression analysis

The raw counts for each gene were calculated by featureCounts [72] at the gene (locus) level and normalized by the gene size and the total number of reads that mapped in the most highly expressed genes, as proposed in the upper quartile (UQ) method described by Bullard et al. [77]. Thus, the raw counts after normalization were called FPKM-UQ (FPKM for Fragment Per Kilobase and Milllions—UQ for Upper Quartile). This method is particularly relevant because highly expressed genes are known to account for most of the reads and therefore to strongly influence the total read number, whereas they represent only a small fraction of the expressed genes. In our study, the top 10 and 25% most highly expressed genes represent 34 and 96% of the reads, respectively, in the liver, and 16 and 90% in the adipose tissue. Finally, a gene was considered as expressed in a tissue when at least 10 of the 16 samples per tissue had a FPKM-UQ greater or equal to 0.1, a threshold often used in studies focusing on lncRNAs [6, 8, 38, 78]. In this study, such a threshold corresponds to eight and two average reads for coding (1987 nt long) and long noncoding (494 nt long) transcripts, respectively. To determine this minimum number of samples (10 of 16) for defining a gene as expressed in one tissue, we analyzed the reproducibility of expression across the 16 biological replicates in each tissue (see the “Results” section and Fig. 1d). Moreover, to provide an estimation of the background signal and then justify the expression threshold of 0.1, we sampled, several times, a set of genomic intervals with the same size distribution as that of our lncRNA loci, and with no overlapping with any gene (protein-coding genes and non-coding genes) using the “bedtools shuffle” command. We refer to this set as the “no-gene” set. We then counted the numbers of reads in these sets for the 16 liver replicates and transformed these read counts into FPKM-UQ (see Additional file 7: Fig. S3). First, we can observe that the third quartile is approximately 0.1 (on the left of Additional file 7: Figure S3). Second, the distribution of the “no gene” set that satisfied the FPKM-UQ threshold of 0.1 across the 16 replicates is very different from those observed for lncRNAs: only 8% of the loci satisfied our double criteria “at least 10 of the 16 samples had a FPKM-UQ greater or equal to 0.1”. Thus, we conclude that our criteria allow us to distinguish expressed entities with a low but reproducible expression from noise with a lower signal that is less reproducible.

For the tissue-specificity analysis, a gene expressed in one tissue was considered as not expressed in the other tissue if its expression was below the FPKM threshold of 0.1 in at least 12 of the 16 samples.

### Co-expression analysis

A lncRNA/protein-coding RNA pair was considered as significantly correlated in a tissue across the 16 replicates when the correlation p value was lower or equal to 0.1 after correction for multiple-testing by the Benjamini–Hochberg method [79]. Pearson correlations were calculated using the log10(FPKM-UQ). For all expressed gene pairs, we considered the highest correlations among those calculated for either liver or adipose tissue. To replicate the analyses with “coding–coding” pairs, we reconstituted “coding–coding” pairs for divergent, convergent and same-strand FEELnc classes in accordance with the FEELnc nomenclature.

### RT-qPCR

Total RNA and cDNA were prepared from various tissues, as previously described by Roux et al. [80]. Four experimental bird designs were analyzed: 16 young males (9 weeks old) analyzed in this study with RNA-Seq data (FatInteger Project—ANR-11-SVS7), 56 adult laying hens (over 30 weeks of age) from the ChickStress Project—ANR-13-ADAP, 20 young males (9 weeks old) fed ad libitum or fasted for 16 h, and finally the multi-tissue design with 17 different tissues, as reported in Roux et al. [80]. RT-qPCR was performed with the primers included in Table 2, and amplification specificity was confirmed by sequencing. The results are given either as CT (cycle threshold) or, for the multi-tissue design, as fold-change compared to a CT equal to 30 (considered as very weak expression).

**Table 2:** RT-qPCR primers used to amplify genes of interest

Gene of interest	Forward	Reverse
DHCR24	TGGAGAGCCCAAAACGAAACA	CGCGGGTCATGTAGCAATC
lncRNA_DHCR24	GAGAGAAGCTGGATGGTCCTG	CTGAAGGAGACTGCAAGGTGT
NPNT	CGATGAATGTGCTACTGGGAGA	AACTACCACACTGATGCTGGC
lncNPNT-AS	TGCACTCTCATCTTGTGTGCT	CAACGTGACCATAAGGGCTG

## Additional files

**Additional file 1: Fig. S1.** Global Jaccard index for our RNA-Seq data calculated with various threshold values using the R software HTSfilter [32]. This figure shows on the left, count data for long noncoding RNAs; and on the right, count data for protein-coding genes. Count data were normalized by TMM methods [81]. For each type of gene, the data-based threshold corresponds to the red cross and red dotted line. For the long noncoding genes (left), the curve shape of the Jaccard index (that gives a threshold—at the maximum of the curve—equal to one read) is not consistent with the expected index curve shape, in contrast to the protein-coding genes (right) that behave correctly, with a maximum of approximately 32 reads.

**Additional file 2: Table S1.** LncRNA transcripts (2412) and lncRNA genes (1670) and their classes according to the FEELnc classification. These lncRNA transcripts and genes are characterized by a good reproducibility of expression in at least one of the two tissues. We provide ID, genomic location, FASTA sequence, length, classification with the closest protein-coding gene and FPKM-UQ mean expression in the liver and adipose tissue. LncRNAs that were also predicted by the CPC method [24] and representing more than 90% of the lncRNAs are indicated in the column CPC.

**Additional file 3: Table S2.** Enriched biological process GO terms of the liver- or adipose-specific subsets for protein-coding genes and long noncoding genes. The enrichment was performed using DAVID software [41, 42], and the GO terms were considered enriched according to the DAVID thresholds used by default.

**Additional file 4: Table S3.** Four subsets of adipose- and liver-specific genes for long noncoding and protein-coding genes.

**Additional file 5: Fig. S2.** Visualization by IGV of the lncLSTR, APOA1-AS and HULC loci in the chicken genome. IGV: Integrative Genomics Viewer from the Broad Institute. LncLSTR [60] was expected between *HMCN1* and *Ivns1abp* genes localized on Scaffold JH375182.1. *HULC* long noncoding gene was expected between the *OFCC1* and *SLC35B3* genes localized on chromosome 2, and *APOA1*-*AS* was expected to overlap with *APOA1* localized on chromosome 24. Expected locations are in *red squares*. The liver RNA-Seq data used here are a merge of the .bam files of the 16 samples. The chicken reference genome was the Ensembl Galgal4, and the annotation version was Ensembl v84.4.

**Additional file 6.** Command lines and input files used to run the different analyses in the current study.

**Additional file 7: Fig. S3.** Boxplot and distribution across the 16 replicates of the “no gene” set representing the noise signal. This figure shows on the left the boxplots of FPKM-UQ for 10 “no gene” sample sets (see “Methods” section) in white compared to those for lncRNAs (red) and mRNAs (blue); and on the right, the distribution across the 16 replicates of the “no gene” set (green) that satisfied the FPKM-UQ threshold of 0.1. This distribution is very different from those observed for lncRNAs (red) or mRNAs (blue).
