# Supplementary figures and images for: Long noncoding RNA repertoire in chicken liver and adipose tissue
Source: Genet Sel Evol. 2017 Jan 10;49:6. doi: 10.1186/s12711-016-0275-0 (PMC5225574; doi:10.1186/s12711-016-0275-0)

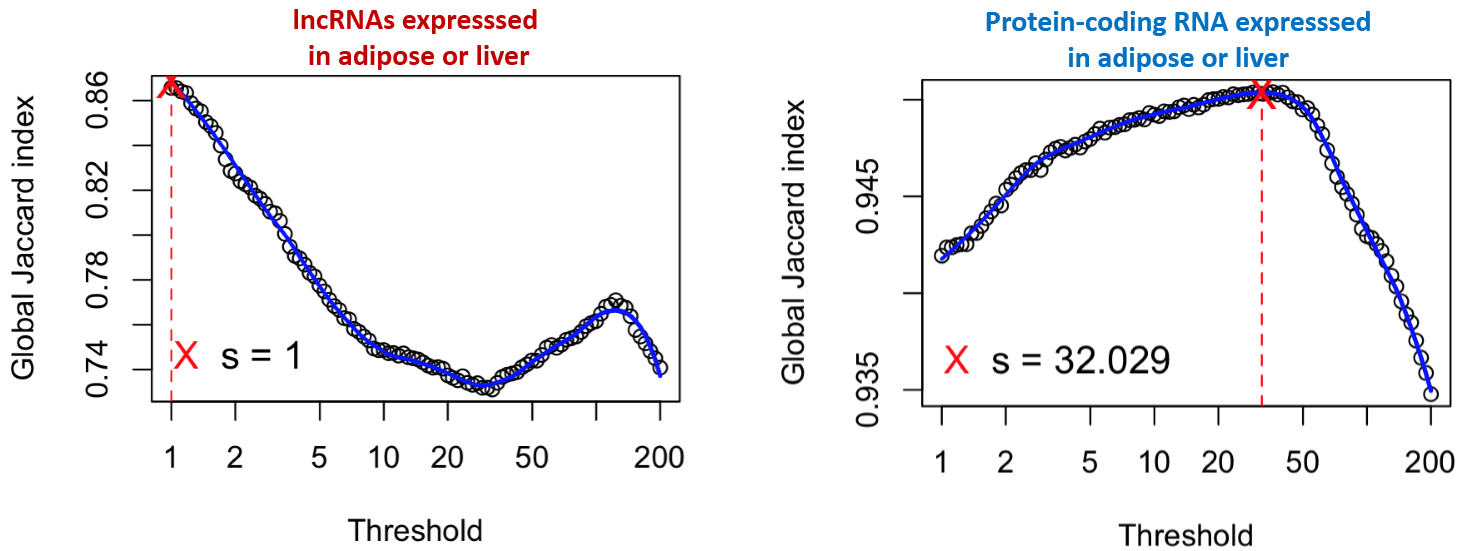

Supplement: Supplementary file 1 — Additional file 1: Fig. S1. Global Jaccard index for our RNA-Seq data calculated with various threshold values using the R software HTSfilter [32]. This figure shows on the left, count data for long noncoding RNAs; and on the right, count data for protein-coding genes. Count data were normalized by TMM methods [81]. For each type of gene, the data-based threshold corresponds to the red cross and red dotted line. For the long noncoding genes (left), the curve shape of the Jaccard index (that gives a threshold—at the maximum of the curve—equal to one read) is not consistent with the expected index curve shape, in contrast to the protein-coding genes (right) that behave correctly, with a maximum of approximately 32 reads. [file 12711_2016_275_MOESM1_ESM.docx]

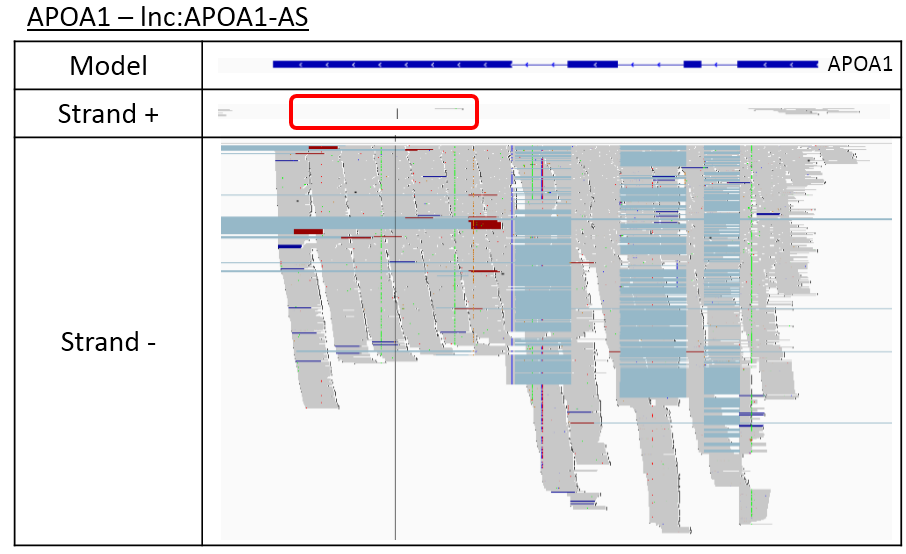


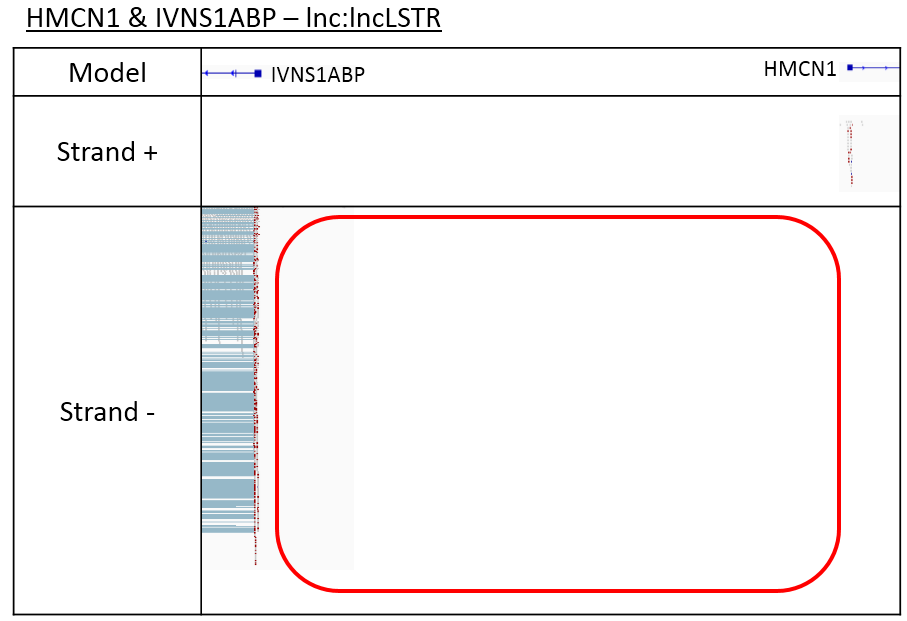


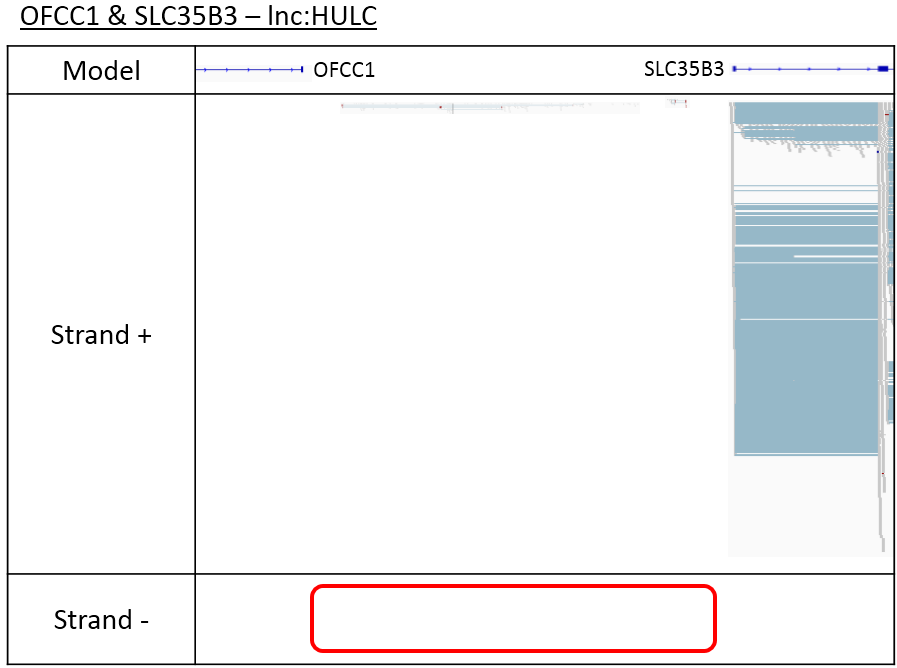

Supplement: Supplementary file 5 — Additional file 5: Fig. S2. Visualization by IGV of the lncLSTR, APOA1-AS and HULC loci in the chicken genome. IGV: Integrative Genomics Viewer from the Broad Institute. LncLSTR [60] was expected between HMCN1 and Ivns1abp genes localized on Scaffold JH375182.1. HULC long noncoding gene was expected between the OFCC1 and SLC35B3 genes localized on chromosome 2, and APOA1-AS was expected to overlap with APOA1 localized on chromosome 24. Expected locations are in red squares. The liver RNA-Seq data used here are a merge of the .bam files of the 16 samples. The chicken reference genome was the Ensembl Galgal4, and the annotation version was Ensembl v84.4. [file 12711_2016_275_MOESM5_ESM.docx]
