# Supplementary material for: Long noncoding RNA repertoire in chicken liver and adipose tissue
Source: Genet Sel Evol. 2017 Jan 10;49:6. doi: 10.1186/s12711-016-0275-0 (PMC5225574; doi:10.1186/s12711-016-0275-0)
Supplement: Supplementary file 6 — Additional file 6. Command lines and input files used to run the different analyses in the current study. [file 12711_2016_275_MOESM6_ESM.docx]

***A.Gene modelling***

***1-For read trimming***

*trim_galore*

*–paired FileR1.fq.gz FileR2.fq.gz*

*# output files => FileR1_val_1.fq.gz FileR2_val_2.fq.gz*

***2-Genome mapping:***

*STAR*

*--runThreadN 8*

*--genomeDir STAR*

*--outStd SAM*

*--outSAMstrandField intronMotif*

*--readFilesIn FileR1_val_1.fq.gz FileR2_val_2.fq.gz*

*--alignIntronMin 10*

*--alignIntronMax 25000*

*--outFilterMismatchNmax 10*

*--outFileNamePrefix File*

*--readFilesCommand zcat |*

*awk '{if($0 ~ /XS:A:/ || $1 ~ /^@/) print $0; else {if(and($2,0x10)==16) print $0"\tXS:A:-"; else print $0"\tXS:A:+";}}' |*

*samtools view*

*-bS*

*-@ 2*

*-o |*

*samtools sort*

*-@ 2*

*-o File.sort*

*# output files => File.sort.bam*

***3-gene modelling***

*samtools rmdup*

*-S File.sort.bam File.rmdup.bam*

*Merge of all .bam files with samtools merge*

*cufflinks*

*-o DIR*

*-p 12*

*-g gga4.gtf*

*--library-type fr-firststrand*

*-I 25000*

*--overlap-radius 5*

*--intron-overhang-tolerance 5*

*-q File.rmdup.bam*

*List of files in a File_list.txt file.*

*cuffmerge*

*-o DIR_Cuffmerge*

*-g gga4.gtf*

*File_list.txt*

*# output files => merged.gtf*

***4-read counting***

*featureCounts*

*-s 2*

*-O*

*-p*

*-t exon*

*-g transcript_id*

*-a merged.gtf*

*-o File.featureCounts*

*File.sort.bam*

*************************************************************************************************

***B. LncRNA prediction by FEELnc***

***1-Transcript filtering***

*FEELnc_filter.pl*

*-i merged.gtf*

*-b transcript_biotype=protein_coding,pseudogene*

*--size=200*

*--monoex=-1*

*--biex=1*

*--proc=6*

*-a gga4.gtf*

*> candidate_lncRNA.gtf*

***2-Coding Potential Score calculation***

*FEELnc_codpot.pl*

*-i candidate_lncRNA.gtf*

*-a gga4.gtf*

*-g gga4.dna.toplevel.fa*

*-l NONCODEv5.fa*

*-k "1,2,3,6,9,12"*

*--outname="lst_lnc"*

*--spethres=0.97,0.97*

*cat lst_lnc.noORF.gtf lst_lnc.lncRNA.gtf > lst_lnc.lncRNA_noORF.gtf*

***3-lncRNA classification***

*FEELnc_classifier.pl*

*-i lst_lnc.lncRNA_noORF.gtf*

*-a gga4.gtf*

*--window=10000*

*--maxwindow=1000000*

*> candidate_lncRNA_classes.txt*
