# Supplementary material for: Long noncoding RNA repertoire in chicken liver and adipose tissue
Source: Genet Sel Evol. 2017 Jan 10;49:6. doi: 10.1186/s12711-016-0275-0 (PMC5225574; doi:10.1186/s12711-016-0275-0)
Supplement: Supplementary file 6 [file 12711_2016_275_MOESM7_ESM.docx]

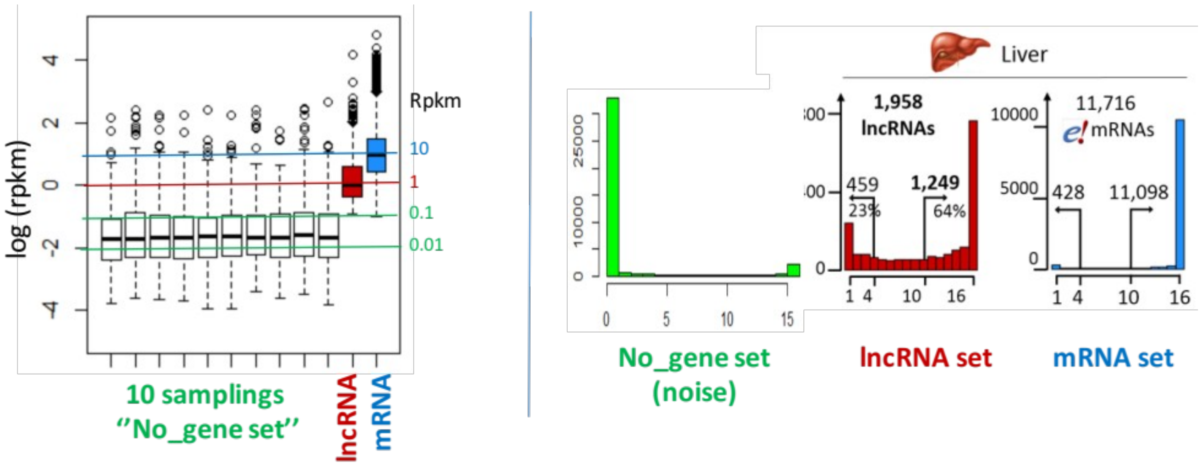

log (rpkm)
Rpkm
10
1
0.1
0.01
10 samplings
"No_gene set"
lncRNA
mRNA
Liver
1,958
lncRNAs
459
23%
1,249
64%
11,716
mRNAs
10000
5000
428
11,098
No_gene set
(noise)
lncRNA set
mRNA set
